# Deficiency of inhibitory TLR4 homolog RP105 exacerbates fibrosis

**DOI:** 10.1172/jci.insight.160684

**Published:** 2022-11-08

**Authors:** Wenxia Wang, Swarna Bale, Bharath Yalavarthi, Priyanka Verma, Pei-Suen Tsou, Ken M. Calderone, Dibyendu Bhattacharyya, Gary J. Fisher, John Varga, Swati Bhattacharyya

**Affiliations:** 1 Northwestern Scleroderma Program, Feinberg School of Medicine, Chicago, Illinois, USA.; 2 Michigan Scleroderma Program, Division of Rheumatology, Department of Internal Medicine, and; 3 Derpartment of Dermatology, University of Michigan, Ann Arbor, Michigan, USA.

**Keywords:** Autoimmunity, Fibrosis

## Abstract

Activation of TLR4 by its cognate damage-associated molecular patterns (DAMPs) elicits potent profibrotic effects and myofibroblast activation in systemic sclerosis (SSc), while genetic targeting of TLR4 or its DAMPs in mice accelerates fibrosis resolution. To prevent aberrant DAMP/TLR4 activity, a variety of negative regulators evolved to dampen the magnitude and duration of the signaling. These include radioprotective 105 kDa (RP105), a transmembrane TLR4 homolog that competitively inhibits DAMP recognition of TLR4, blocking TLR4 signaling in immune cells. The role of RP105 in TLR4-dependent fibrotic responses in SSc is unknown. Using unbiased transcriptome analysis of skin biopsies, we found that levels of both TLR4 and its adaptor protein MD2 were elevated in SSc skin and significantly correlated with each other. Expression of RP105 was negatively associated with myofibroblast differentiation in SSc. Importantly, RP105-TLR4 association was reduced, whereas TLR4-TLR4 showed strong association in fibroblasts from patients with SSc, as evidenced by PLA assays. Moreover, RP105 adaptor MD1 expression was significantly reduced in SSc skin biopsies and explanted SSc skin fibroblasts. Exogenous RP105-MD1 abrogated, while loss of RP105 exaggerated, fibrotic cellular responses. Importantly, ablation of RP105 in mice was associated with augmented TLR4 signaling and aggravated skin fibrosis in complementary disease models. Thus, we believe RP105-MD1 to be a novel cell-intrinsic negative regulator of TLR4-MD2–driven sustained fibroblast activation, representing a critical regulatory network governing the fibrotic process. Impaired RP105 function in SSc might contribute to persistence of progression of the disease.

## Graphical abstract

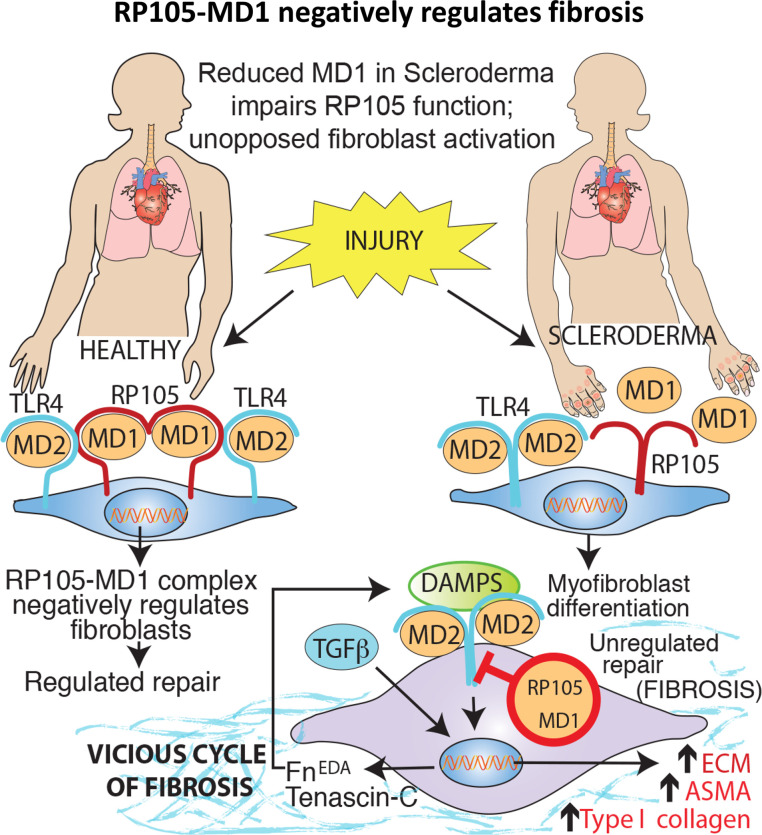

## Introduction

The innate immune system recognizes both pathogen-derived molecule patterns (PAMPs) from Gram-negative bacteria (LPS) and damage-associated molecular patterns (DAMPs) generated in situ as a result of chronic injury via pattern recognition receptors such as TLR4 (1, 2). TLR4 comprises an extracellular leucine-rich repeat ligand-binding domain and an intracellular signaling Toll/IL-1 receptor homology (TIR) domain. Upon binding of ligand, the extracellular leucine-rich repeat region of TLR4 forms a complex with myeloid differentiation factor 2 (MD2), followed by dimerization and conformational changes of the intracellular TIR domains, leading to recruitment and activation of adaptor molecules myeloid differentiation primary response 88 (MyD88) and TIR domain–containing adapter-inducing interferon-β (TRIF), which then bind to the intracellular TIR domains to initiate cellular signaling (3–5). We have previously demonstrated that DAMPs such as fibronectin-EDA (Fn-EDA) and tenascin-C are markedly upregulated in skin and lungs, and are markers of tissue fibrosis, in patients with systemic sclerosis (SSc) (6, 7). In mice, genetic ablation of either TLR4 or its cognate DAMPs enhanced fibrosis resolution, suggesting of a fundamental pathogenic role for the DAMP/TLR4 axis in fibrosis persistence or progression (6–8).

Radioprotective 105 kDa (RP105, also known as CD180) is closely related to TLR4 and a transmembrane protein expressed primarily in monocytes and macrophages, dendritic cells, and B cells (9). While similar to TLR4, RP105 lacks the intracytoplasmic TIR domain of TLR4 and is therefore unable to elicit intracellular signaling (9). RP105 function depends on the coexpression of its adaptor protein MD1; thus, it resembles the overall architecture of the TLR4-MD2 complex. Crystal structure analysis revealed a novel RP105-MD1 tetrameric complex of 2 RP105 and 2 MD1 molecules. In contrast to the DAMP-TLR4-MD2 complex, RP105 and MD1 are organized in a unique head-to-head arrangement mode, in which leucine-rich repeat modules of 2 RP105 chains place their N-terminal regions in the center of the complex, while simultaneously interacting with 2 MD1 molecules. This arrangement of RP105-MD1 juxtaposes with the tail-to-tail dimerization of TLR4-MD2 that is highly conserved in ligand-activated TLR homo- and heterodimers, thus competitively blocking TLR4 binding to its ligands (9, 10). The activities of RP105-MD1 on regulating TLR4-driven responses are cell-type dependent (11). For instance, while in B cells RP105/MD1 functions as a stimulator of LPS-induced NF-κB activation (12), whereas in dendritic cells and macrophages, RP105/MD1 attenuated LPS-dependent TLR4 responses (9, 13).

Excessive accumulation of collagen and other extracellular matrix proteins in the skin and other critical organs is the hallmark of SSc (14, 15). Differentiation of tissue-resident quiescent fibroblasts and various mesenchymal progenitor cell types into myofibroblasts accounts for the persistence of fibrosis (16–19). Despite the importance of DAMP/TLR4 signaling in SSc and the role of RP105 in modulating TLR4 activity, nothing is currently known regarding RP105 expression and function in stromal cells or its potential pathogenic role in fibrosis. Therefore, this study was undertaken to elucidate the expression and role of RP105 in fibrosis in SSc.

Here, we show that while levels of both MD2 and TLR4 were significantly increased, and positively correlated with each other, in SSc skin biopsies, RP105 showed negative correlation with ASMA expression in SSc skin biopsies as well as in fibroblasts explanted from patients with SSc. Importantly, RP105-TLR4 demonstrated weak association in contrast to strong TLR4-TLR4 association in fibroblasts from patients with SSc, as revealed by proximity ligation assays (PLAs). Furthermore, MD1 showed significantly reduced expression in skin biopsies and skin fibroblasts from patients with SSc. Ectopic RP105-MD1 blocked TLR4-induced profibrotic responses, while RP105-deficient fibroblasts showed augmented fibrotic responses that were abrogated by a small-molecule TLR4 inhibitor. Importantly, mice with global loss of function of RP105 demonstrated exaggerated skin inflammation and fibrosis in complementary mouse models. Thus, we believe RP105-MD1 to be a novel cell-intrinsic negative regulator of DAMP-TLR4–dependent progressive fibrosis. Compromised RP105-MD1 function might contribute to the disease progression in SSc.

## Results

### RP105 levels negatively correlated with ASMA levels in SSc skin biopsies and explanted fibroblasts.

**Figure 1:**
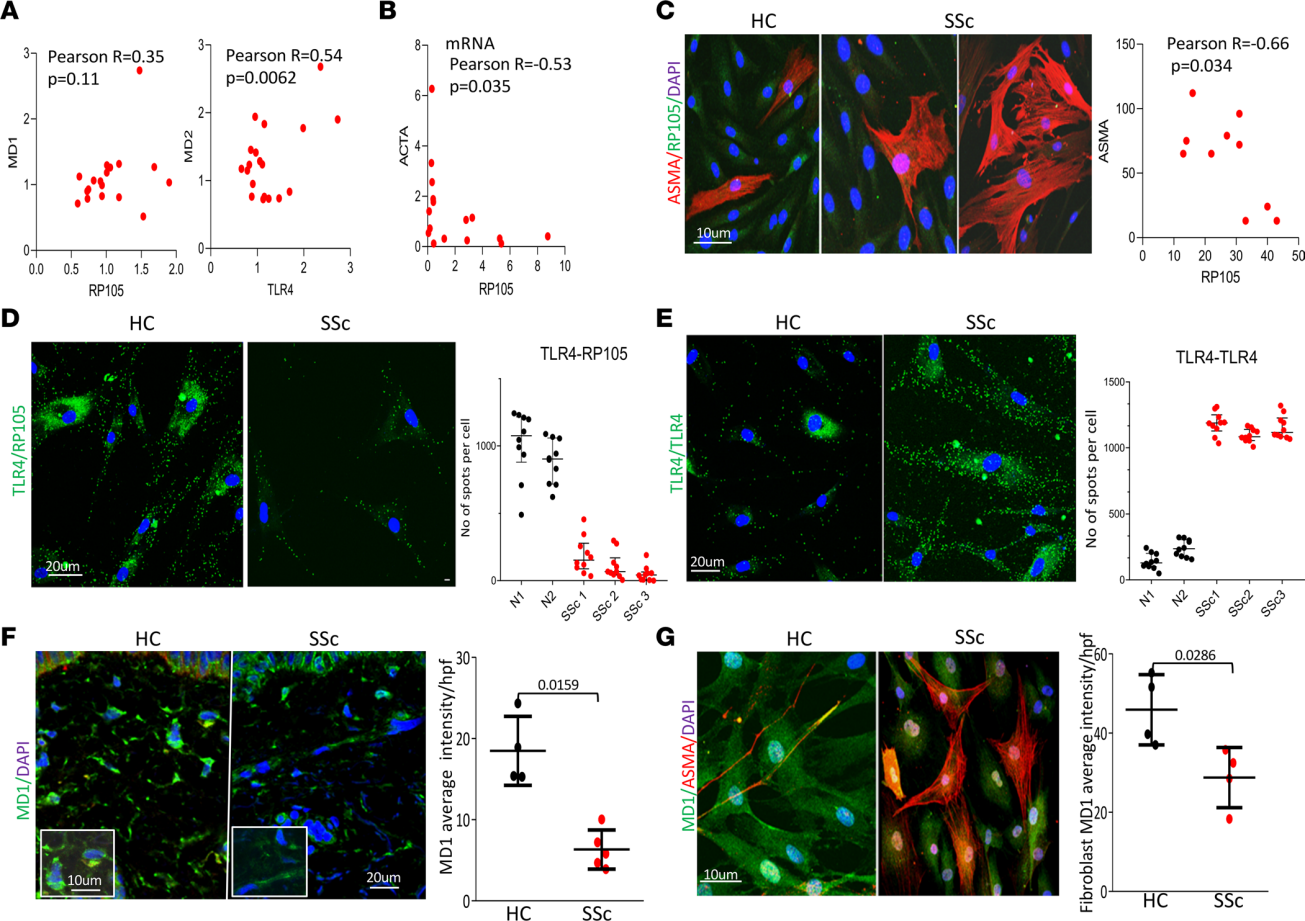
RP105 negatively correlated with ASMA and expression of its adaptor MD1 was reduced in SSc. (**A**) Correlation of MD1 with RP105 levels and MD2 with TLR4 levels from publicaly available SSc transcriptome data sets (GSE32413), representing skin biopsies from 22 patients with dcSSc and 9 healthy controls. (**B**) Correlation of RP105 with ASMA mRNA levels using SSc (*n* = 16) and healthy controls fibroblasts (*n* = 6). Pearson’s correlation coefficient. (**C**) Skin fibroblasts from SSc (*n* = 10) and healthy control (*n* = 5) were immunolabeled using antibodies against RP105 and ASMA (left panel). Each dot represents a single biopsy (right panel) and represent the mean fluorescent intensity of both RP105 and ASMA was measured in the same cell from 3 different fields containing at least 3–4 cells/hpf. Representative images are shown. Scale bar: 10 μm (left panel). Pearson’s rank correlation (right panel). (**D** and **E**) Results from PLA assays using explanted SSc and healthy control skin fibroblasts. (**D**) RP105-TLR4 and (**E**) TLR4-TLR4 PLA signals (**F** and **G**, left panels) and PLA quantification (**D** and **E**, right panels). Blue, DAPI; green, PLA signal. Mann-Whitney *U* test. Representative images are shown. Scale bar: 20 μm. (**F**) Skin biopsies from SSc (*n* = 5) and healthy control (*n* = 4) were immunolabeled using antibodies against MD1. Representative images are shown (left panel). Quantitative data using SSc and healthy control skin biopsies were immunolabeled using antibodies against MD1. Average intensity from 3 cells/hpf from 4 different areas per skin biopsy. Mann-Whitney *U* test. Scale bar: 10 μm; 20 μm (inset). (**G**) Skin fibroblasts from SSc (*n* = 4) and healthy control (*n* = 4) were immunolabeled using antibodies against MD1 and ASMA (left panel). Each dot represents a single biopsy (right panel) and represent mean fluorescent intensity of MD1 from 3 different fields containing at least 3–4 cells/hpf from the indicated number of participants. Mann-Whitney *U* test. Representative images are shown. Scale bar: 10 μm; 20 μm (SSc). Each dot represents a single biopsy.

In light of the pathogenic role of aberrant DAMP/MD2/TLR4 signaling in persistent fibrosis in SSc (18, 19), we sought to characterize the expression of TLR4 and its unique coreceptors MD2 and MD1 in SSc. For this purpose, we interrogated publicly available SSc transcriptome data sets (GSE32413) that represent skin biopsies from a total of 22 patients with diffuse cutaneous SSc (dcSSc) and 9 healthy controls. While *TLR4* and *MD2* mRNA expression showed significant positive correlation with each other (*r* = 0.54, *P* = 0.0062, Pearson’s correlation), *RP105* and *MD1* had no significant association (*r* = 0.35, *P* = 0.11, Pearson’s correlation) (Figure 1A) (20). There was no significant association between RP105 and MD1 expression with modified Rodnan skin score, a marker of SSc disease severity (RP105, *r* = –0.16, *P* = 0.43 and MD1, *r* = –0.10, *P* = 0.63, respectively). Similarly, RP105 showed significant negative correlation with expression of *ACTA* (ASMA) in fibroblasts from patients with SSc at both mRNA (*r* = –0.53, *P* = 0.035; Pearson’s correlation) (Figure 1B) and protein (*r* = –0.66, *P* = 0.034; Pearson’s correlation) levels (Figure 1C). Similarly, RP105 expression was reduced in ASMA^+^ myofibroblasts, as shown by double immunofluorescence using healthy and SSc skin biopsies (*r* = –0.38, *P* = 0.04; Pearson’s correlation), without showing any significant difference in the expression levels between healthy controls and patients with SSc (Supplemental Figure 1; supplemental material available online with this article; https://doi.org/10.1172/jci.insight.160684DS1). Importantly, PLA assays exhibited weak RP105-TLR4 fluorescence signals in explanted fibroblasts from patients with SSc compared with those from healthy controls (Figure 1D). Conversely, strong TLR4-TLR4 PLA association signals were detected in patients with SSc compared with healthy controls (Figure 1E). Moreover, MD1 showed reduced expression in SSc skin biopsies (*P* = 0.015) and explanted SSc skin fibroblasts (*P* = 0.028) compared with those from healthy controls (Figure 1, F and G). The clinical features of the patients are shown in Table 1.

### Ectopic RP105-MD1 attenuated TLR-dependent fibrotic cellular responses elicited by PAMP and DAMP.

**Figure 2:**
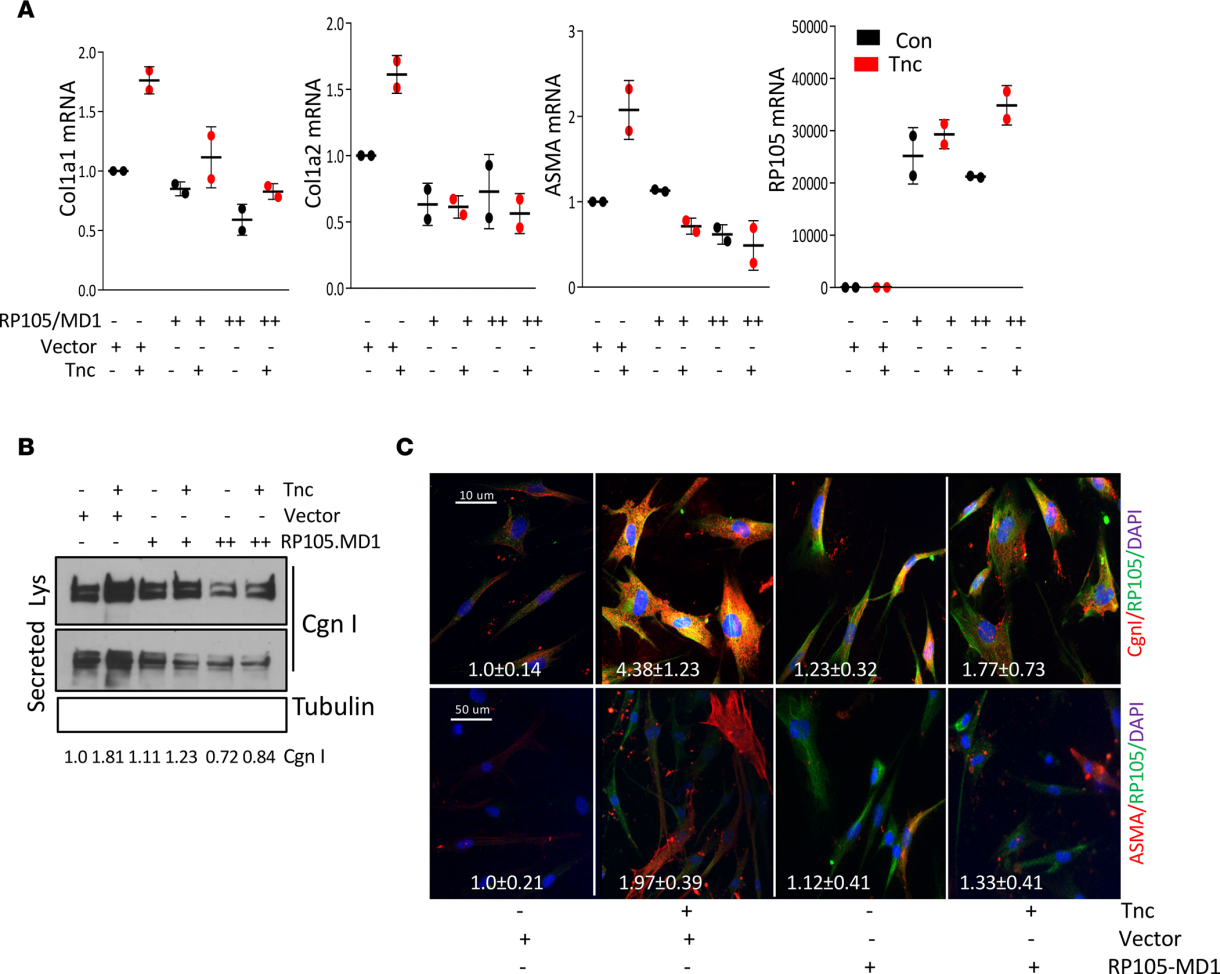
RP105 inhibited TLR4-dependent profibrotic responses. Human foreskin fibroblasts were transfected with RP105/MD1 duo plasmid and control plasmid (100 or 250 ng) followed by incubation with tenascin-C (2 μg/ml) for 72 hours. (**A**) Real-time quantitative PCR expressed as fold change compared with vector control. Results, normalized with GAPDH, are shown as the mean ± SD of triplicate determinations from 2 separate experiments. (**B**) Whole-cell lysates examined by Western analysis. Representative immunoblots are shown. Type I collagen levels, normalized for tubulin, are shown below. (**C**) Immunofluorescence confocal microscopy. Representative images are shown. Scale bar: 50 μm.

Activation of fibroblasts by the prototypic TLR4 ligand (LPS) or by endogenous DAMP ligands elicits potent profibrotic responses (14, 18, 19, 21). In a variety of cells, RP105 negatively regulates TLR4-dependent inflammatory responses. To assess the potential role of RP105 in modulating profibrotic responses, we pursued complementary approaches using human skin fibroblasts. We previously showed that both PAMPs and DAMPs elicited TLR4-dependent upregulation of extracellular matrix protein remodeling– and fibrosis-related genes (6–8). To examine how RP105-MD1 might modulate TLR4-mediated profibrotic signaling, human skin fibroblast cultures were incubated with LPS or tenascin-C in the presence of RP105 and MD1. Remarkably, ectopic RP105-MD1 expression potently attenuated DAMP-dependent profibrotic responses and myofibroblasts differentiation (Figure 2).

**Figure 3:**
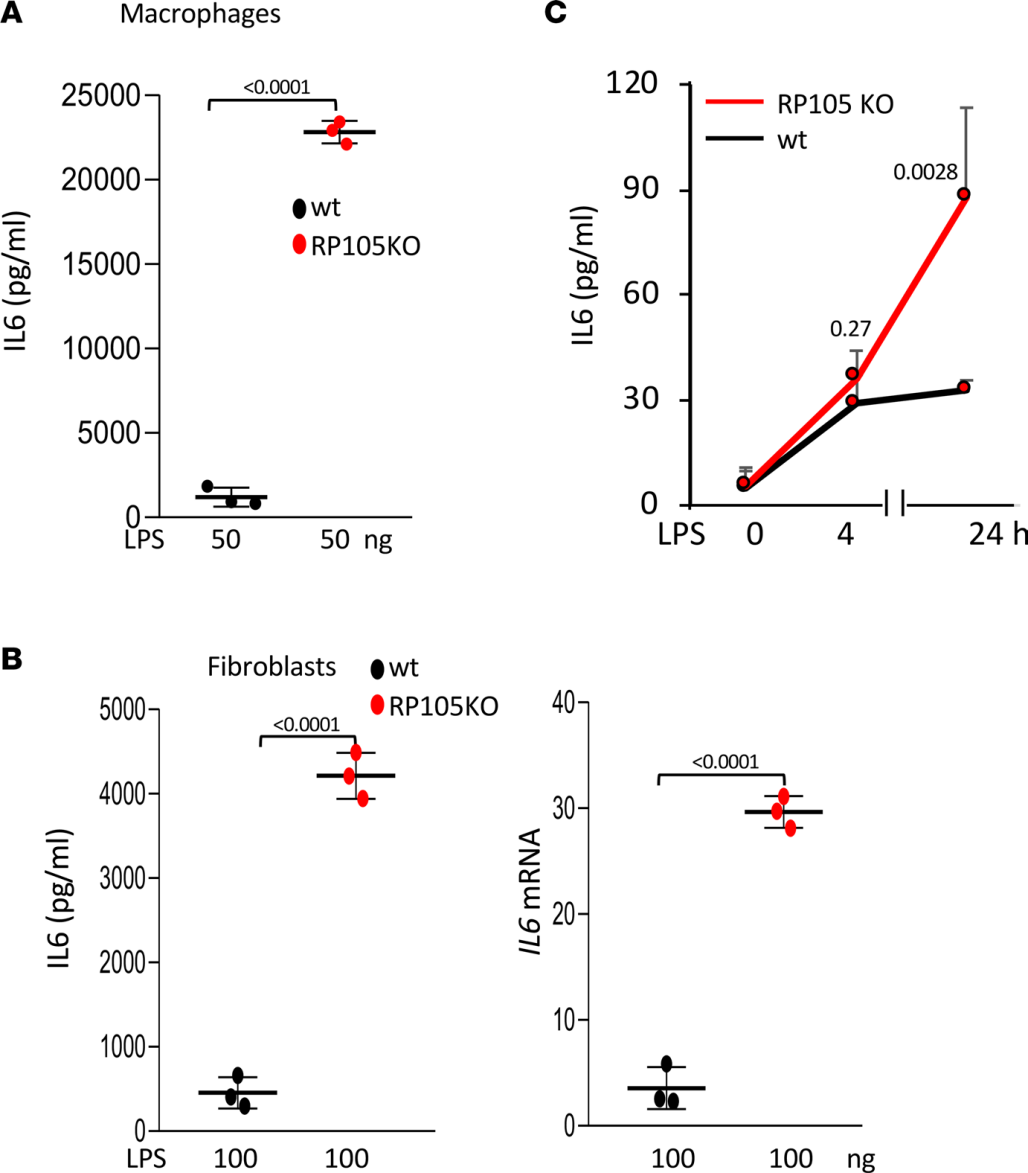
RP105 negatively regulates immune and stromal cell TLR4 responses. (**A**) Bone marrow–derived macrophages and (**B**) skin fibroblasts were isolated from 8-week-old WT and RP105-KO mice. Cultures were incubated in media with or without LPS at indicated concentrations, and after 24 hours, IL-6 concentration in blood and secreted in media and cellular RNA were examined by (**A** and **B**, left, and **C**) ELISA or (**B**, right) qPCR analysis. Results are shown as the mean ± SD of triplicate determinations from 3 mice/group. The quantitative concentrations of IL-6 are plotted and compared with a standard curve by ELISA from secreted media from macrophages and fibroblasts and from the whole blood ex vivo assay. The control and LPS-treated WT and KO IL-6 levels were plotted. For qPCR, results, normalized with GAPDH, are shown as the mean ± SD of triplicate determinations expressed as fold change (control and LPS-treated WT and control and KO-treated group fold-change values were compared). *P* values were determined by unpaired 2-tailed Student’s *t* test. (**C**) Whole blood was collected from RP105-KO and C57/BL6 mice and incubated with LPS for indicated periods. Sera were examined by ELISA. Results are shown as the mean ± SD of triplicate determinations from 3 mice/group.

**Figure 4:**
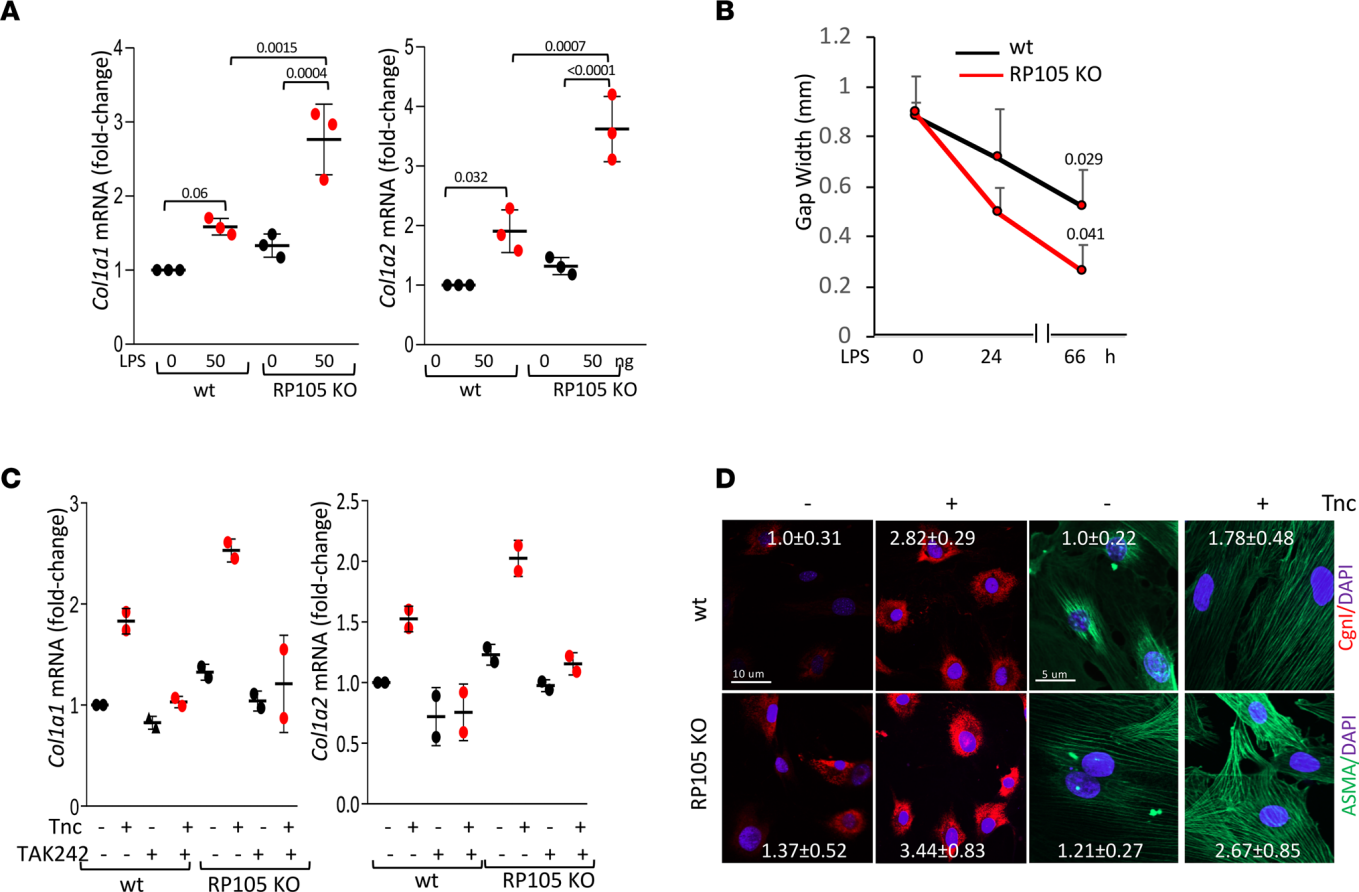
Lack of RP105 accentuates TLR4-dependent profibrotic responses. (**A** and **B**) Skin fibroblasts explanted from RP105-KO and C57/BL6 mice were incubated in the presence or absence of LPS. (**A**) Real-time quantitative PCR. Results, normalized with GAPDH, are shown as the mean ± SD of triplicate determinations. One-way ANOVA followed by Šidák’s multiple comparisons test. (**B**) In vitro wound healing assays. Results are shown as the mean ± SD of triplicate determinations in 4 randomly selected fields. Mann-Whitney *U* test. (**C** and **D**) Skin fibroblasts were incubated with tenascin-C in presence of TAK242 for 72 hours. (**C**) Real-time quantitative PCR. Results, normalized with GAPDH, are shown as the mean ± SD of triplicate determinations. One-way ANOVA followed by Šidák’s multiple comparisons test. (**D**) Immunostaining using antibodies against CgnI and ASMA. Nuclei were identified by DAPI (blue). Representative confocal microscopic images. Scale bar: 10 μm (red); 5 μm (green). Quantification of immunofluorescence (inset). The results shown the mean ± SD from 3 randomly selected hpf/slide.

We next compared the effect of RP105 on TLR4-dependent signaling in different immune and stromal cell populations using cells explanted from RP105-KO and age-matched WT mice. Both bone marrow–derived macrophages and skin fibroblasts from RP105-deficient mice showed significantly accentuated proinflammatory LPS responses compared with WT cells (*P* < 0.0001) (Figure 3, A and B). Furthermore, LPS incubation of whole blood from RP105-KO mice ex vivo resulted in significantly higher IL-6 levels compared with similarly treated blood from WT mice (*P* = 0.0028) (Figure 3C). Remarkably, RP105-deficient skin fibroblasts in vitro showed significantly greater profibrotic gene expression, collagen accumulation, ASMA expression, and cell migration upon a scratch injury, compared with WT fibroblasts (Figure 4, A, B, and D). Very importantly, treatment with a small-molecule TLR4 inhibitor, TAK242, attenuated the profibrotic responses in RP105-KO fibroblasts as well as WT fibroblasts (Figure 4C). These complementary experiments with mouse and human fibroblasts collectively implicate RP105 as a potent cell-intrinsic negative regulator of TLR4-dependent profibrotic signaling.

### RP105 ablation augmented skin fibrosis in mice.

**Figure 5:**
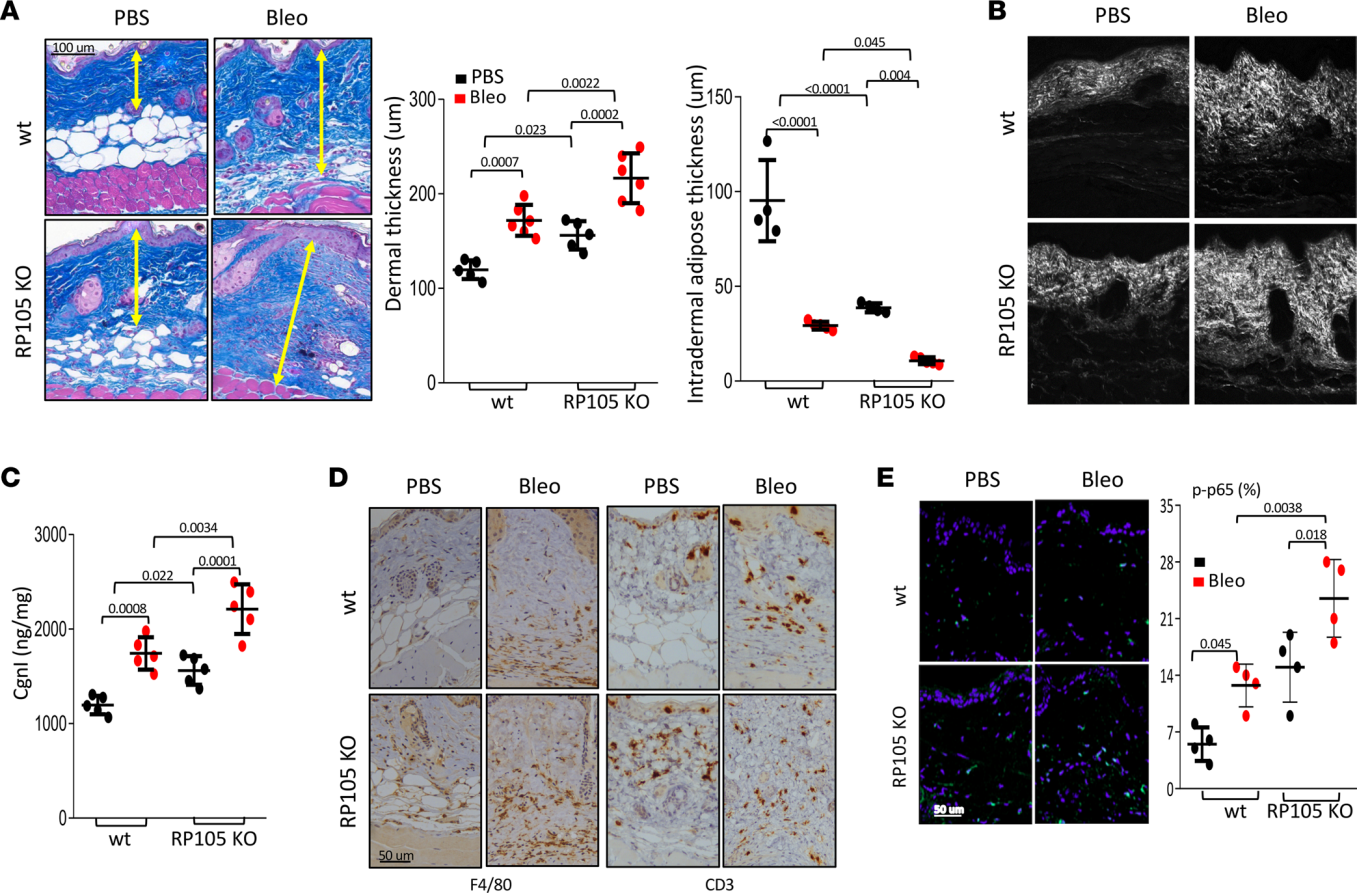
RP105-KO mice showed exaggerated skin fibrosis. Eight- to 12-week-old female RP105-KO and WT mice received daily s.c. injections of bleomycin or PBS for 2 weeks (5 days per week) and were sacrificed on day 22, when the skin was harvested for analysis. (**A**) Trichrome stain (yellow arrows indicate dermal thickness). Representative images are shown. Scale bar: 100 μm. Dermal thickness (mean ± SD of 8 determinations/hpf; middle). Intradermal adipose thickness (mean ± SD of 8 determinations/hpf; right). One-way ANOVA followed by Šidák’s multiple comparisons test. (**B**) Second-harmonic generation microscopy images of dermal collagen fibrils. Representative images are shown (*n* = 12 mice, 3 for each group). For scale bars, the original scan dimensions were 387.65 microns square. (**C**) Collagen content. Dots represent the mean ± SD from duplicate determination from the indicated number of mice. One-way ANOVA followed by Šidák’s multiple comparisons test. (**D**) Immunolabeling with antibodies against F4/80 and CD3 and F4/80. Representative images are shown. Scale bar: 50 μm. (**E**) Immunofluorescence using antibodies against phospho-p65 (left). Scale bar: 50 μm. Quantitation of immunopositive cells (number/hpf from 4 different areas from each skin section, *n* = 4 mice). One-way ANOVA followed by Šidák’s multiple comparisons test.

To examine the in vivo contribution of RP105 in fibrosis, we evaluated the effect of RP105 deletion in preclinical disease models. First, we compared fibrosis induced by s.c. bleomycin injected daily for 2 weeks (5 days per week) in RP105-KO and WT mice. Remarkably, we observed that, even in the absence of bleomycin treatment, PBS-injected RP105-KO mice showed increased dermal fibrosis associated with loss of s.c. adipose tissue (Figure 5, A and B). Furthermore, significant increases of F4/80^+^ myeloid cells (*P* = 0.025) and CD3^+^ T cells (*P* = 0.015) were noted in PBS group skin (Figure 5D and Supplemental Figure 2A). Bleomycin treatment accentuated skin fibrosis in RP105-KO mice (Figure 5, A–C) and was associated with increased accumulation of F4/80^+^ cells surrounding remnants of intradermal adipocytes (*P* = 0.0059), with a concomitant increase in ASMA (Supplemental Figure 2, A and B). Further examination of the skin by second-harmonic generation microscopy showed well-organized collagen fibers throughout the dermis in WT mice and increased collagen fiber deposition in RP105-KO mice. Bleomycin treatment further enhanced collagen fibril deposition in RP105-KO mice, compared with that in WT mice (Figure 5B). Importantly, the skin from RP105-KO mice showed increased numbers of phospho-p65^+^ cells (*P* = 0.0038) (Figure 5E) as well as a consistent and significant increase in accumulation of the TLR4 endogenous ligand Fn-EDA (*P* = 0.025) with bleomycin treatment (Supplemental Figure 2C), an indicator of activated TLR4 signaling. Tenascin-C, a similar TLR4 ligand, was inconsistently elevated in RP105-KO mice (Supplemental Figure 2C). Bleomycin treatment significantly augmented inflammatory expression of genes, such as *Il1b* and *Il*6, in RP105-KO mouse skin (Supplemental Figure 3). Intriguingly, 6-month-old RP105-KO mice showed a spontaneous fibrotic phenotype, with s.c. adipose tissue atrophy and increased dermal thickness (*P* = 0.029), F4/80^+^ cell accumulation, and collagen deposition (*P* = 0.028) (Supplemental Figure 4).

### RP105 ablation enhanced inflammation-independent fibrosis.

**Figure 6:**
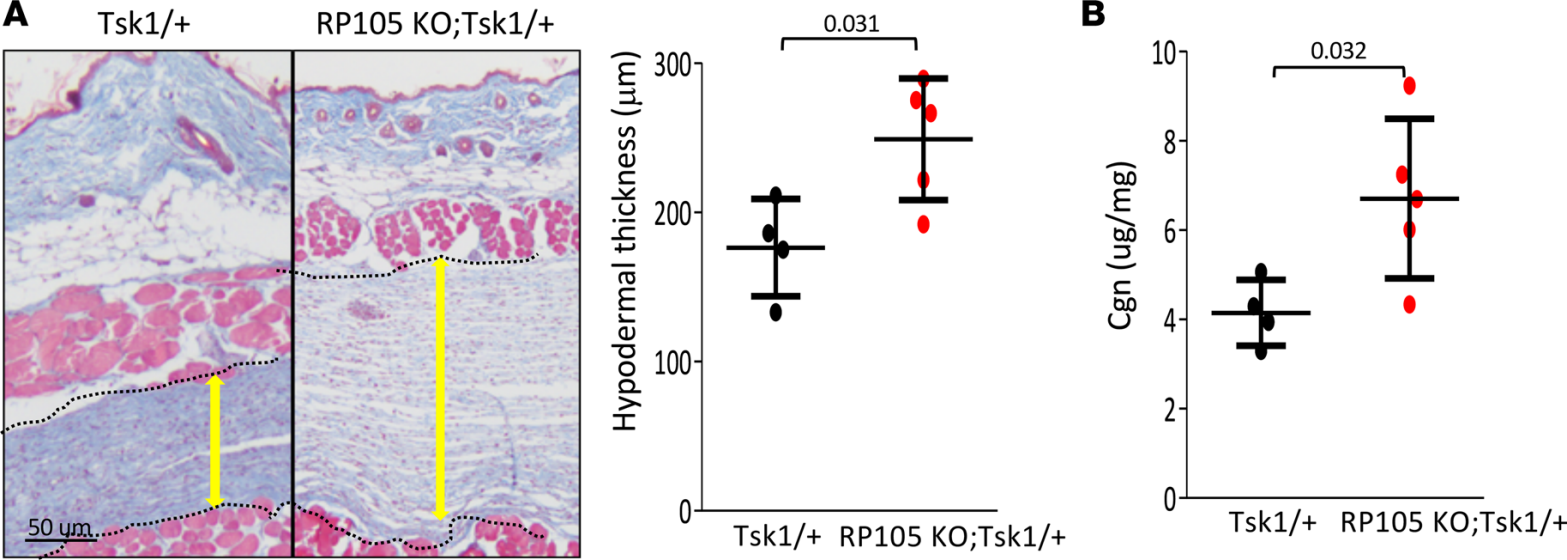
RP105-KO; Tsk1/+ mice showed exaggerated spontaneous skin fibrosis. Twelve-week-old RP105-KO; Tsk1/+ and Tsk1/+ transgenic mice were sacrificed, and skin was harvested for analysis. (**A**) Trichrome stain (left). Representative images are shown (dotted lines indicate hypodermis and yellow arrows show hypodermal thickness). Hypodermal thickness (right) (mean ± SD of 8 determinations/hpf). Scale bar: 50 μm. Student’s *t* test. (**B**) Collagen content of the skin. Dots represent the mean ± SD from duplicate determinations from the indicated number of mice. One-way ANOVA followed by Šidák’s multiple comparisons test.

To explore the role of RP105 in a noninflammatory model of skin fibrosis, we generated RP105-KO; Tsk1/+ transgenic mice, because TSK/+ mice have fibrotic features without prominent inflammatory features. Due to a duplication mutation in FBN1, Tsk1/+ mice spontaneously develops fibrosis of the skin (22). Moreover, Tsk1/+ mice show increased deposition of endogenous TLR4 ligands in the skin (6). At 12 weeks of age, compared with Tsk1/+ mice, RP105-KO; Tsk1/+ mice showed significantly increased hypodermal thickening (*P* = 0.031) (Figure 6A) and increased collagen accumulation (*P* = 0.032), without any noticeable difference in inflammation (Figure 6B). Together, these results demonstrate that RP105 regulates fibrotic responses in both inflammatory and noninflammatory models of fibrosis.

## Discussion

Although several pathways capable of promoting fibroblast activation in SSc have been identified, their interplay in the context of fibrosis persistence and progression remains incompletely understood (14, 23). Emerging evidence suggests a role for innate immunity and pattern recognition signaling in wound healing and fibrosis (21, 24, 25). We had previously shown that endogenous TLR ligand DAMPs trigger TLR4-mediated fibrosis amplification loops, resulting in progression of fibrosis (20, 21). Conversely, genetic ablation of TLR4 and its DAMPs or treatment with selective TLR4 inhibitors ameliorates experimental skin fibrosis (6–8, 18). Alternatively, impaired negative regulation of TLR4 signaling in SSc might result in unrestrained TLR4-dependent progression of fibrotic responses in SSc. The present study focused on the TLR4-inhibitory coreceptor RP105 in fibrosis. We show that ectopic RP105 inhibited both TLR4 PAMP- or DAMP-induced profibrotic responses in fibroblasts. Mice lacking RP105 showed enhanced spontaneous and inducible skin fibrosis and inflammation accompanied by increased TLR4 signaling and DAMP accumulation; explanted RP105-KO fibroblasts showed elevated profibrotic and proinflammatory TLR4 responses.

TLR4 activation by LPS or endogenous DAMPs requires accessory adaptor MD2. Inhibition of TLR4/MD2 activity by RP105-MD-1 involves formation of a TLR4-MD2/RP105-MD1 complex, resembling the usual ligand-induced TLR homodimers (26). While our results provide evidence for augmented MD2 and TLR4 expression and association, RP105 and MD1 showed none of the significant association that is required for their proper functioning. Formation of the unusual complex by TLR4-MD2 and RP105-MD1 due to lack of adequate RP105/MD1 in SSc might results in unopposed TLR4 signaling. Lack of RP105 expression in ASMA-expressing fibroblasts corroborated our findings that RP105 deficiency is leading myofibroblast transformation and sustained activation underlying intractable skin fibrosis. Importantly, reduced MD1 in SSc skin biopsies and fibroblasts might further facilitate TLR4-MD2 complex formation in SSc, promoting fibrosis progression. This result is consistent with earlier data that shows reduced MD1 expression in dilated cardiomyopathy (27). Furthermore, loss of MD1 was associated with several cardiovascular diseases; it aggravates maladaptive left atrial fibrosis, ischemia/reperfusion injury, ischemia/reperfusion-related arrhythmia, and inflammation through enhanced activation of the TLR4 signaling pathway (28–31). Importantly, we showed that, in contrast to robust TLR4-TLR4 PLA signals, weak RP105-TLR4 showed weak PLA signals in SSc. This observation further strengthens our hypothesis that in SSc reduced MD1 has prevented the association of MD1 with RP105 and absence of the RP105-MD1 complex facilitates TLR4-TLR4 complex formation and fibrosis progression.

Here, we used complementary gain-of-function and loss-of-function approaches to decipher the potential role of RP105-MD1 in fibrosis. Our results support our hypothesis that ectopic RP105-MD1 mitigated TLR4-dependent profibrotic responses and loss of RP105 augmented both inflammatory and profibrotic processes in vitro and in vivo. Underlying mechanisms might involve increased TLR4/NF-κB signaling, possibly as a result of augmented inflammatory responses and the accumulation of profibrotic DAMPs. Indeed, we found a consistent increase in TLR4 DAMP Fn-EDA in RP105-KO mice skin.

RP105-KO skin fibroblasts showed augmented profibrotic responses in addition to enhanced TLR4 responses in fibroblasts and myeloid cells. Importantly, TLR4 inhibitor TAK242 attenuated TLR4-driven profibrotic responses in both WT and RP105-KO skin fibroblasts, further strengthening the fact that the antifibrotic effect of RP105 is primarily accomplished by inhibiting TLR4 signaling. However, the results were based on in vitro and ex vivo studies. It would be interesting to examine the effect of TAK242 in vivo in RP105-KO mice and precisely determine the fibroblast-specific role of RP105, which is beyond the scope of this current study and represents one of the limitations of this study.

Although RP105 is uniquely important for regulating TLR4-dependent signaling, it has a context-specific function. For instance, in myocardial ischemia/reperfusion injury, RP105 plays a cardioprotective role by modulating both TLR2/TLR4 signaling pathways (32). A recent report implicated RP105 in preventing renal ischemic and septic acute kidney injury mediated by TLR4 signaling pathways (33). Cell-type-specific differences can also determine RP105 activity. Unlike myeloid cells and fibroblasts, in B cells TLR4 and RP105 showed LPS-induced NF-κB activation, implying functional cooperation in this context (12). Nevertheless, our in vivo study using RP105-KO mice showed TLR4-dependent exaggerated skin fibrosis, confirming RP105 as a negative regulator of DAMP/TLR4 signaling. Targeting of the TLR4/MD2 signaling complex by RP105-MD1 resulted in inhibition of proinflammatory as well as profibrotic responses elicited by endogenous TLR4 DAMPs. Future studies will be required to determine the role of RP105 in other forms of fibrosis and explore the possibility that targeting (inducing) RP105 will have a protective role in fibrosis.

## Methods

### Cell culture and reagents.

Primary cultures of fibroblasts were established by explanations from SSc and healthy adult skin, neonatal foreskin, and newborn mouse skin from WT and RP105-KO mice. Briefly, the skin biopsies were digested using 0.2% collagenase (Sigma-Aldrich, C0130), in DMEM with 20% FBS at 37°C for 3 hours, centrifuged, and washed; the skin tissue was resuspended with DMEM with 20% FBS. The skin tissue was transferred gently into 100 mm sterile-treated Petri dishes, followed by scraping off the epidermis using sterile scalpel. Next, the tissue was chopped into fine pieces, and explanted fibroblasts were allowed to grow in DMEM with 20% FBS. The media was replaced until the explanted fibroblasts were confluent. Low-passage fibroblasts were grown in monolayers in plastic dishes and studied at early confluence. Cultures were maintained in Dulbecco’s Modified Eagle’s medium supplemented with 10% fetal bovine serum (Gibco BRL), 1% vitamin solutions, and 2 mM glutamine. All other tissue culture reagents were from Lonza. For experiments, cultures were placed in serum-free media containing 0.1% bovine serum albumin overnight, followed by treatment with LPS (InvivoGen), TGF-β1 (Peprotech), or human Tenascin-C (Sigma-Aldrich) or transfection with pDUO-MD1/RP105 (InvivoGen) for up to 72 hours.

To generate bone marrow–derived macrophages and dendritic cells, femurs and tibias from WT (C57BL/6J) and RP105-KO mice were harvested and cultured as described previously (34, 35). To evaluate the expression of IL-6 on macrophages, dendritic cells, and whole blood, cells and whole blood were incubated with LPS at indicated concentrations for 24 hours. IL-6 expression from the culture supernatants and mice sera were assayed using an ELISA (R&D Systems, catalog M6000B).

### Isolation and analysis of RNA.

At the end of the experiments, total RNA was isolated and reverse transcribed to cDNA using Supermix (Quanta Biosciences, cDNA Synthesis Supermix) as described previously (18). The products (20 ng) were amplified using SYBR Green PCR Master Mix (Applied Biosystems) on an Applied Biosystems 7500 Prism Sequence Detection System. Data were normalized to GAPDH RNA, and fold change in samples was calculated. Sequences of the primers are shown in Table 2.

### Cell migration assay.

The effect of RP105 on fibroblast function was further evaluated by an in vitro wound-healing assay. Briefly, human skin fibroblasts from both WT and RP105-KO mice were seeded on serum-free DMEM, and confluent monolayers were mechanically wounded using p1000 pipette tips. Following incubation of the cultures with TGF-β for indicated periods, wound gap widths (μm) were determined at indicated time points at 6 randomly selected sites per high-powered field (hpf). The experiments were performed in 2 different skin fibroblasts lines.

### Western analysis.

At the end of the experiments, cultures were harvested and washed with PBS, followed by addition of RIPA lysis buffer mixed with phosphatase and protease inhibitors. Cells were lysed and spun down and were subjected to protein estimation bicinchoninic acid assay using bovine serum albumin as standard for comparison. Then, equal volumes of cell lysates (20 μg/lane) were denatured and subjected to Western analysis using primary antibodies specific for type I collagen (Southern Biotechnology) and tubulin (Sigma-Aldrich, T5168), followed by appropriate secondary antibodies as described previously (18). Membranes were then exposed to enhanced chemiluminescence detection using ECL Reagent (Pierce). Band intensities were quantitated using Image J software (NIH) and corrected for tubulin in each lane.

### Transfection study.

Subconfluent fibroblast cultures were transfected with pDUO-MD1/RP105 (InvivoGen) using Lipofectamine reagent (Thermo Fisher Scientific), followed by tenascin-C treatment for 72 hours. Cultures were harvested, and whole-cell lysates were assayed for Western analysis (6). The experiment was repeated twice with consistent results.

### PLA.

The PLA was carried out using Sigma Duolink reagents (Sigma-Aldrich, DUO92009). and fibroblasts from healthy controls and patients with SSc were cultured in 8-well chamber slides, fixed in ice-cold methanol for 5 minutes, and subjected to blocking using Duolink blocking solution (Sigma-Aldrich, DUO92009) for 1 hour at 37°C in a humidity chamber. For TLR4 homodimerization assay, anti-TLR4 (mouse, Invitrogen, PA5-23124, 1:200) and ant-TLR4 (rabbit, Santa Cruz, sc293072, 1:100) were used, while for testing RP105-TLR4 dimerization, anti TLR4 (mouse, Invitrogen, PA5-23124, 1:200) and anti-RP105 (rabbit, Abcam, ab184956, 1:100) were used. Slides were incubated overnight at 4°C in the primary antibodies diluted in Duolink antibody diluent (Sigma-Aldrich) followed by washing twice with (5 minutes) in 1× Wash Buffer A (Sigma-Aldrich) at room temperature. Slides were then further incubated with PLA probe solution composed of PLA plus and minus probes (Sigma-Aldrich) diluted in the Duolink antibody diluent. Incubation was carried out in a preheated humidity chamber for 1 hour at 37°C. Slides were washed twice for 5 minutes in 1× Wash Buffer A (Sigma-Aldrich, DUO82049-4L) and subjected to ligation reaction in a preheated humidity chamber for 30 minutes at 37°C, followed by washing twice with for 5 minutes in 1× Wash Buffer A (Sigma-Aldrich) at room temperature, and subjected to amplification reaction in a preheated humidity chamber in the dark for 100 minutes at 37°C. Slides were washed in the dark twice for 10 minutes in 1× Wash Buffer B (Sigma-Aldrich, DUO82049-4L), followed by a 1-minute wash in 0.01× Wash Buffer B. Slides were mounted with Duolink In Situ Mounting Medium with DAPI (Sigma-Aldrich, DUO82040-5ML). Slides were imaged using confocal microscopy. For quantitation, raw format confocal image files were split into individual channels by ImageJ. The green channel was subjected to thresholding. In the binary image, individual cells were selected, and spot numbers were calculated using analyze particle tool with a minimum size of 0.4 μm.

### Experimental models of skin fibrosis.

Complementary fibrosis models were used to evaluate the effect of RP105-KO mice from Christopher L. Karp (Cincinnati Children’s Hospital, Cincinnati, Ohio, USA) in vivo. First, 8-week-old female C57BL/6J mice received daily s.c. injections of bleomycin (10 mg/kg/d) or PBS for 2 weeks (5 days per week) and were sacrificed on day 22; lesional skin was harvested for analysis. The experiments were repeated 2 times with consistent results. In a complementary experiment, a noninflammatory fibrosis model was used. Eight-week-old female Tsk1/+ mice (C57BL/6 background, The Jackson Laboratory) were crossed with RP105-KO mice to generate RP105-KO; Tsk1/+ mice. At 12 weeks of age, RP105-KO; Tsk1/+ and Tsk/+ mice were sacrificed, and lesional skin was harvested for analysis. Six-month-old female C57BL/6J and RP105-KO mice were sacrificed, and skin was harvested for analysis (6, 36).

Paraffin-embedded tissue sections (4 μm) were stained with hematoxylin and eosin or trichrome. The thickness of the dermis and hypodermis, defined as the distance from the epidermal-dermal junctions to the dermal-adipose junction or to the loose connective tissue subjacent to the panniculus carnosus, respectively, were determined at 5 randomly selected sites/hpf (37).

For immunohistochemistry, sections of paraffin-embedded skin were immunolabeled with primary antibodies against F4/80 (Invitrogen, 1:500, 14-4801-82), CD3 (Abcam, 1:3000, ab16669), and ASMA (Abcam, 1:500, ab5694), followed by appropriate biotinylated secondary antibodies (Jackson Immunoresearch, 1:250), and detected using biotin complex conjugated with horseradish peroxidase (Vector Laboratories) and 3,3′-diaminobenzidine (DAB; Dako) for color development. The collagen content of the skin was determined by hydroxyproline assays (Biovision, Colorimetric Assay Kits). Total RNA isolated from mouse skin biopsies was reverse transcribed to cDNA using Supermix and analyzed by real-time qPCR (Applied Biosystems) on an Applied Biosystems 7500 Prism Sequence Detection System as described previously (37).

### Immunofluorescence confocal microscopy using skin fibroblasts and skin biopsies.

Fibroblasts seeded on 8-well Lab-Tek II chamber glass slides (Nalgene Nunc International) were incubated in serum-free DMEM with or without tenascin-C (2 μg/ml) for 72 hours. Cells were then fixed, permeabilized, and incubated with antibodies against ASMA (Abcam, 1:500, ab5694), type I collagen (Southern Biotechnology, 1:100, 1310-01), RP105 (Abcam, 1:100, ab184956), and MD1 (Santa Cruz, 1:50, sc-390613), followed by Alexa Fluor–labeled secondary antibodies (Invitrogen) as described previously (18). For tissue immunofluorescence analyses, paraffin-embedded skin sections were incubated with ASMA (Abcam, 1:100, ab5694), p-p65 (Cell Signaling Technology, 1:100, 3033), Fn-EDA (Abcam, 1:100, ab6328), tenascin-C (Abcam, 1:100, ab108930), RP105 (Abcam, 1:100, ab184956), and MD1 (Santa Cruz, 1:50, sc-390613), followed by appropriate secondary antibodies. Nuclei were detected using DAPI. Slides were mounted, and immunofluorescence and immunohistochemistry were evaluated under Nikon A1R laser scanning confocal microscope in a blinded manner. Negative controls stained without primary antibodies were used to confirm immunostaining specificity.

### Second-harmonic generation.

Imaging of dermal collagen fibers by second-harmonic generation was performed as previously described (38). Briefly, paraffin-embedded skin sections (12 μm thick) were scanned on a Leica SP8 microscope equipped with a Coherent Chameleon MP laser. SHG emissions were elicited using 2-photon excitation at 820 nm and detected through 410/10 nm bandwidth filters. Forward and backward SHG signals were detected by externally mounted photomultiplier tubes. Image stacks were 3D Gaussian blurred and flattened using maximum projection in ImageJ (FIJI) (39).

### Statistics.

Mann Whitney *U* test and 2-tailed Student’s *t* test were used for comparisons between 2 groups, with *P* < 0.05 considered statistically significant. Comparisons among 3 or more groups were performed using 1-way ANOVA followed by Šidák’s correction for multiple comparisons. Pearson’s rank correlations were calculated to measure the correlation. Data are presented as mean ± SD unless otherwise indicated. Data were analyzed using Graph Pad Prism (version 8).

### Study approval.

Biopsies were performed with written informed consent and following protocols approved by the Institutional Review Board for Human Studies at Northwestern University and the University of Michigan. Animal experiments were performed according to protocols approved by Northwestern University and the University of Michigan and in compliance with the guidelines of the Northwestern University Animal Care and Use Committee.

## Author contributions

S. Bhattacharyya designed the experiments. WW, S. Bale, PV, BY, DB, PST, and KMC performed experiments. S. Bhattacharyya, KMC, S. Bale, BY, and DB analyzed the data. GJF and JV provided intellectual input and feedback on the manuscript. S. Bhattacharyya prepared the manuscript.

## Supplementary Material

Supplemental data

## Figures and Tables

**Table 1:**
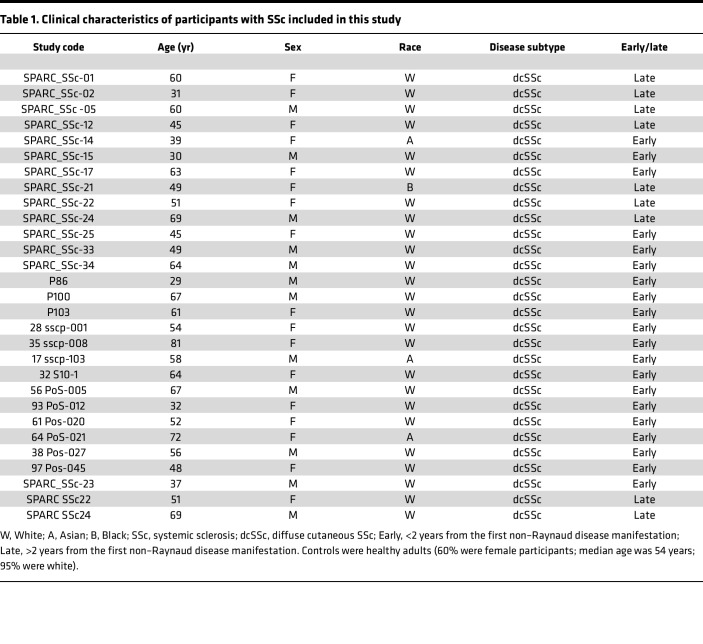
Clinical characteristics of participants with SSc included in this study

**Table 2:**
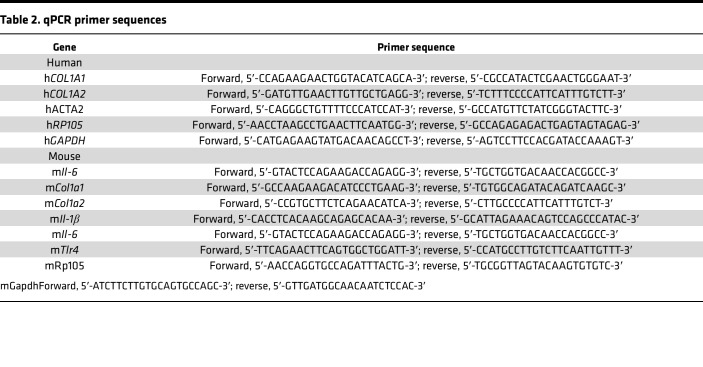
qPCR primer sequences

## Version 1 09/22/2022

In-Press Preview

## Version 2 11/08/2022

Electronic publication
