# Supplementary material for: Deficiency of inhibitory TLR4 homolog RP105 exacerbates fibrosis
Source: JCI Insight. 2022 Nov 8;7(21):e160684. doi: 10.1172/jci.insight.160684 (PMC9675479; doi:10.1172/jci.insight.160684)
Supplement: Supplemental data [file jciinsight-7-160684-s193.pdf]

Supplementary Fig. 1: RP105 negatively correlates with ASMA in SSc and healthy control skin biopsies. A. Skin biopsies from SSc (n=7) and healthy controls (n=4) were immunolabeled using antibodies to RP105 (green) and ASMA (red). Representative immunofluorescence images. Dotted line showed the epidermis and dermis intersection. Scale bars: 100 μm, 20 μm, and 5 μm. **B.** Quantitative data represent the mean fluorescent intensity of both RP105 and ASMA measured in same cell from three different fields containing at least 3-4 cells/hpf from the indicated number of subjects. Pearson's rank correlation.

Supplementary Fig. 2: RP105 KO mice showed increased accumulation of leukocytes and myofibroblasts in the skin. RP105 KO mice and wild-type mice received daily s.c. injections of bleomycin or PBS for two weeks (5 days/week). Mice were sacrificed on day 22, and skin was harvested for analysis. **A.** Immunolabelling. Quantitation showing numbers of F4/80-positive cells /hpf (left panel) and CD3-positive cells /hpf (right panel) from four different areas from each skin section from four mice. One-way ANOVA followed by Sidak's multiple comparison test. **B.** Immunohistochemistry using antibodies to ASMA (left panel). Quantitation of immunohistochemistry images represented as the number of positive cells /hpf from four different areas from each skin section of three mice (right panel). One-way ANOVA followed by Sidak's multiple comparison test. **C.** Immunolabelling using antibodies to Fn-EDA and tenascin-C**.** Representative images. Bar=100 μm.

Supplementary Fig. 3: RP105 KO mice showed increased proinflammatory gene expressions.

RP105 KO and wild-type mice received daily s.c. injections of bleomycin or PBS for two weeks (5 days/week). Mice were sacrificed on day 22, and RNA isolated from skin was subjected to qPCR analysis. Results, normalized with GAPDH, represent the mean ± SD of triplicate determinations from four mice. One-way ANOVA followed by Sidak's multiple comparison test.

Supplementary Fig. 4: Spontaneous skin fibrosis in RP105 KO mice.

At 6 months of age, female RP105 KO and wild-type mice were sacrificed, and skin tissues were harvested for analysis. **A.** Left panel, H&E stain. Representative images. Bar = 100 µm. Right panel, dermal thickness (means ± SD of eight determinations/hpf from the indicated number of mice). The black arrow shows dermal thickness. Mann-Whitney *U* test. **B.** Immunolabelling with antibodies to F4/80. **C.** Skin collagen content. Dots represent the means ± s.d. from duplicate determination from the indicated number of mice**.** One-way ANOVA followed by Sidak's multiple comparison test. Bar=100 µm.

Suppl Fig. 1

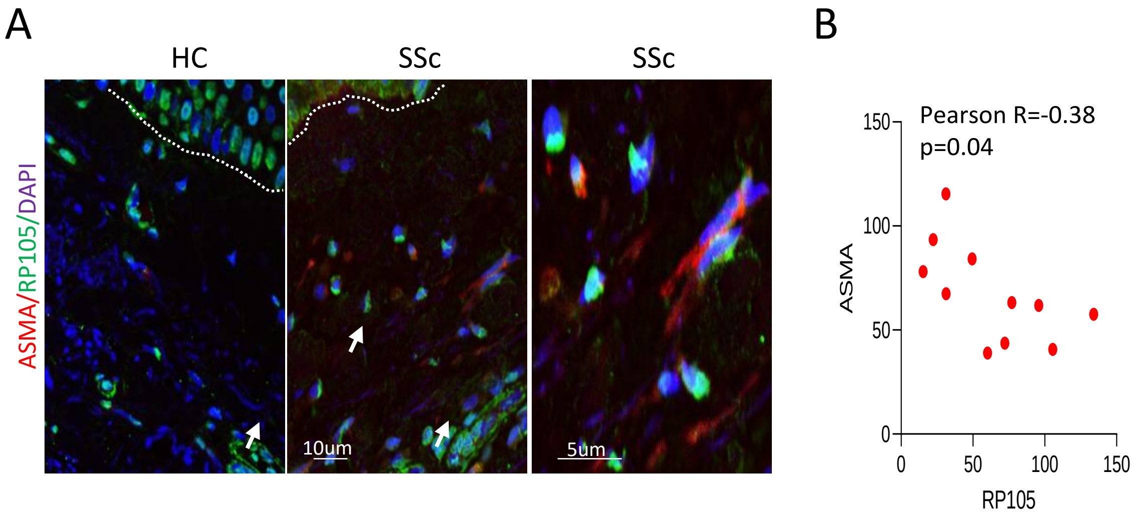
A
HC
SSc
SSc
ASMA/RP105/DAPI
10um
5um
B
Pearson R=-0.38
p=0.04
ASMA
RP105
150
100
50
0
0
50
100
150

Suppl Fig. 2

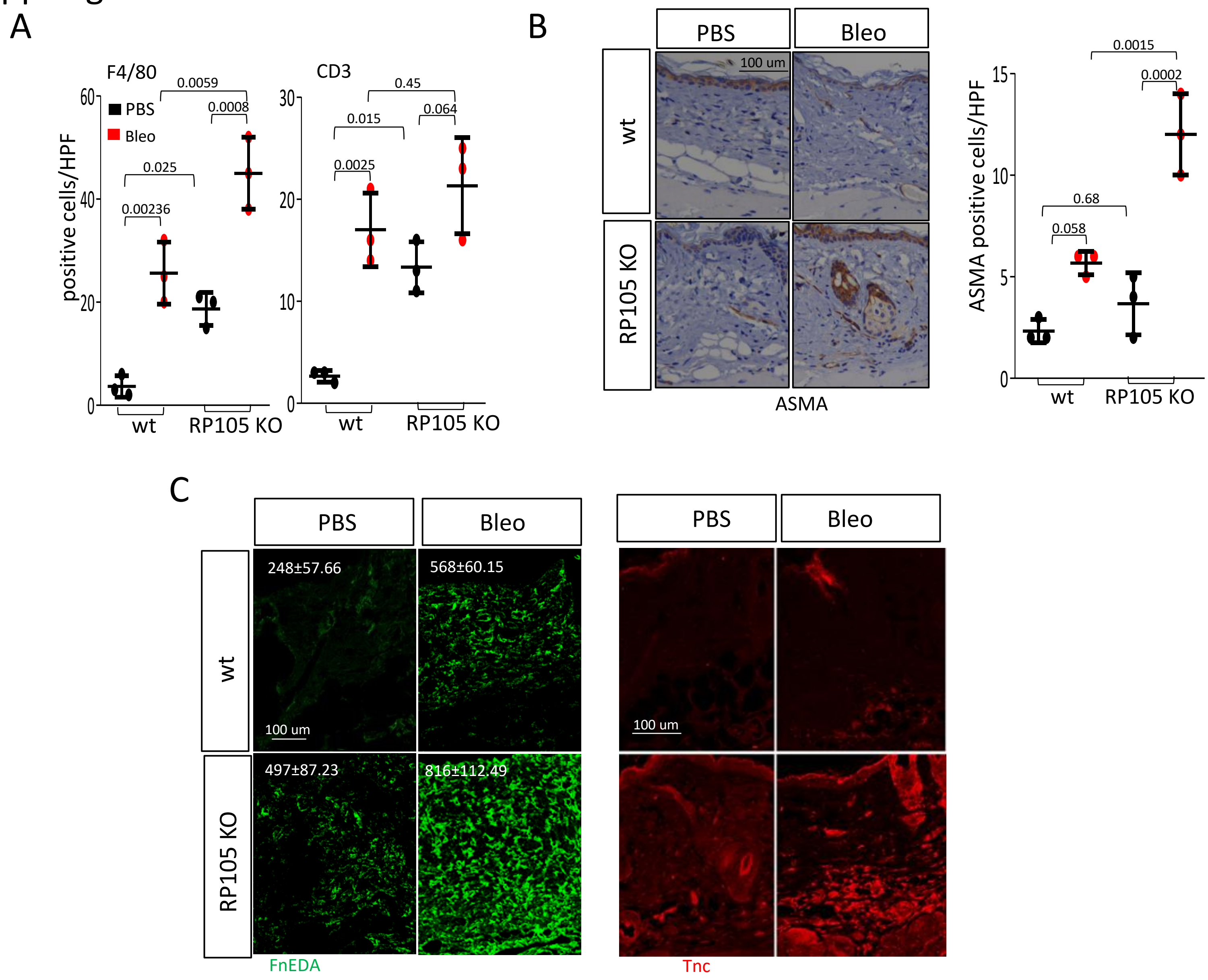

A
F4/80
CD3
PBS
Bleo
positive cells/HPF
0.0059
0.0008
0.025
0.00236
0.45
0.015
0.064
0.0025
wt
RP105 KO
B
PBS
Bleo
wt
RP105 KO
100 um
ASMA
ASMA positive cells/HPF
0.0015
0.0002
0.68
0.058
C
248±57.66
568±60.15
497±87.23
816±112.49
100 um
FnEDA
Tnc

Suppl Fig. 3

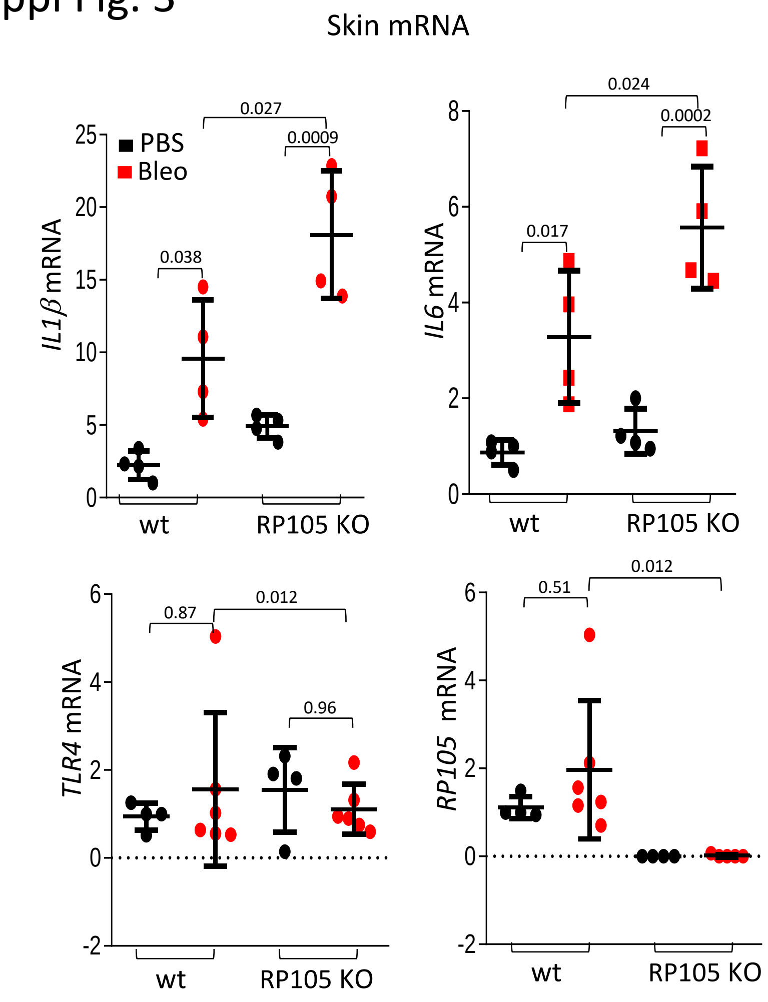

Skin mRNA
PBS
Bleo
IL1β mRNA
IL6 mRNA
TLR4 mRNA
RP105 mRNA
0.027
0.0009
0.038
0.024
0.0002
0.017
0.87
0.012
0.96
0.51
0.012
wt
RP105 KO

Suppl. Fig. 4

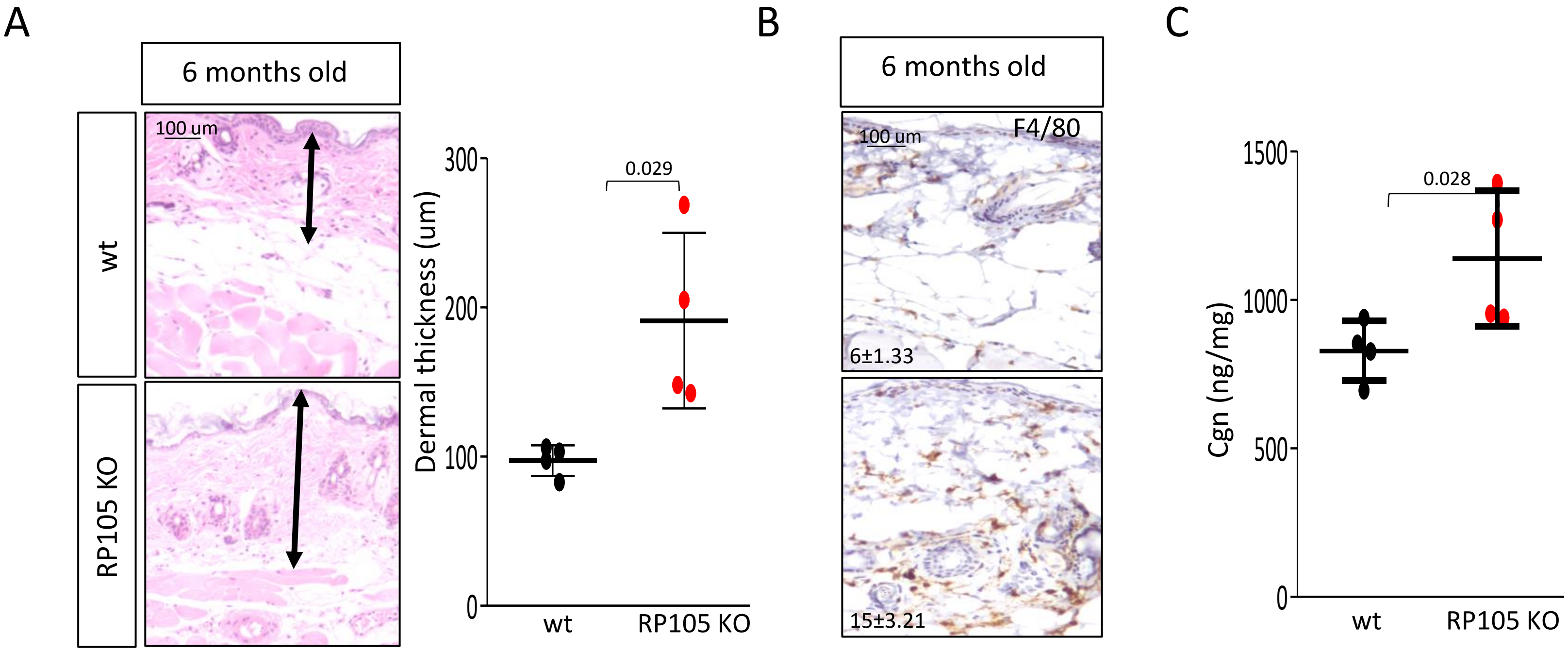
A
6 months old
wt
RP105 KO
100 um
Dermal thickness (um)
300
200
100
0
0.029
wt
RP105 KO
B
6 months old
100 um
F4/80
6±1.33
15±3.21
C
Cgn (ng/mg)
1500
1000
500
0
0.028
wt
RP105 KO
